# Addressing COVID‐19 Drug Development with Artificial Intelligence

**DOI:** 10.1002/aisy.202000070

**Published:** 2020-04-27

**Authors:** Dean Ho

**Affiliations:** ^1^ The N. 1 Institute for Health (N. 1) National University of Singapore Singapore 117456 Singapore; ^2^ The Institute for Digital Medicine (WisDM) Yong Loo Lin School of Medicine National University of Singapore Singapore 117456 Singapore; ^3^ Department of Biomedical Engineering NUS Engineering National University of Singapore Singapore 117583 Singapore; ^4^ Department of Pharmacology Yong Loo Lin School of Medicine National University of Singapore Singapore 117600 Singapore; ^5^ Smart Systems Institute National University of Singapore Singapore 119613 Singapore

**Keywords:** artificial intelligence, combination therapy, COVID-19, drug development, severe acute respiratory syndrome coronavirus 2

## Abstract

The emergence of severe acute respiratory syndrome coronavirus 2 (SARS‐CoV‐2), the virus that led to the COVID‐19 (Coronavirus Disease 2019) pandemic, has resulted in substantial overburdening of healthcare systems as well as an economic crisis on a global scale. This has in turn resulted in widespread efforts to identify suitable therapies to address this aggressive pathogen. Therapeutic antibody and vaccine development are being actively explored, and a phase I clinical trial of mRNA‐1273 which is developed in collaboration between the National Institute of Allergy and Infectious Diseases and Moderna, Inc. is currently underway. Timelines for the broad deployment of a vaccine and antibody therapies have been estimated to be 12–18 months or longer. These are promising approaches that may lead to sustained efficacy in treating COVID‐19. However, its emergence has also led to a large number of clinical trials evaluating drug combinations composed of repurposed therapies. As study results of these combinations continue to be evaluated, there is a need to move beyond traditional drug screening and repurposing by harnessing artificial intelligence (AI) to optimize combination therapy design. This may lead to the rapid identification of regimens that mediate unexpected and markedly enhanced treatment outcomes.

## Graphical abstract

Multiple strategies are being explored to address severe acute respiratory syndrome coronavirus 2 (SARS‐CoV‐2), the virus that causes COVID‐19 (Coronavirus Disease 2019). These include the development of vaccines, antibodies, and combination therapies. Amidst the many clinical trials underway to validate these approaches, artificial intelligence may serve as a foundation for systematically optimized drug development that can markedly enhance treatment outcomes.

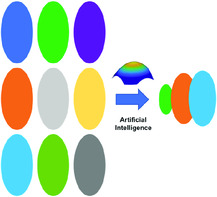

## 1 Challenges with Traditional Combination Therapy and Drug Repurposing

Given the aforementioned timelines to realizing novel drugs/vaccines, drug repurposing particularly as combination therapies has been perhaps the most widely explored among the arsenal of available treatments. In times of urgency, administering drugs that are approved for human use based on the mechanisms of drug action that may also effectively treat COVID‐19 allows for a rapid response. Multiple therapies are currently being studied (Table 1). An open label, individually randomized and controlled trial (NCT02845843) was conducted comparing a combination regimen composed of ritonavir and lopinavir (Kaletra) against the standard of care therapy (e.g., supplemental oxygen, ventilation, antibiotics, and other approaches). The study revealed no benefit of ritonavir/lopinavir administration over the standard of care treatment, and the authors discussed the possibility of combining this regimen with additional therapies to further assess the effectiveness of treatment.^1^ A trial to assess a combination of hydroxychloroquine and azithromycin is underway (NCT04321278). Remdesivir (Gilead Sciences), an investigational therapy previously developed for Ebola, is currently being investigated via two regimens in a phase 3 clinical trial, with the primary outcome being the normalization of oxygen saturation and temperature from first dose to day 14. A secondary outcome is assessing adverse events from first dose to day 10 that may lead to treatment discontinuation (NCT04292899).

**Table 1:** Examples of single/combination drug trials to address COVID‐19

Drug/combination	Clinicaltrials.gov registry number
Hydroxychloroquine, azithromycin, and tocilizumab	NCT04332094
Oseltamivir, favipiravir, hydroxychloroquine, darunavir, lopinavir	NCT04303299
Favipiravir combined with tocilizumab	NCT04310228
Lopinavir/ritonavir, ribavirin, and IFN‐beta combination	NCT04276688
Anakinra, siltuximab, and tocilizumab	NCT04330638
Losartan (nonhospitalized patients)	NCT04311177
Baricitinib and ritonavir	NCT04320277
Emtricitabine/tenofovir and hydroxychloroquine	NCT04334928
Lopinavir/ritonavir or hydroxychloroquine comparison	NCT04307693
Remdesivir	NCT04292899

Aside from these studies, a number of trials are being run to assess other repurposed drug combinations. These range from favipiravir in combination with tocilizumab (NCT04310228); lopinavir/ritonavir, ribavirin, and interferon‐beta (NCT04276688); hydroxychloroquine, azithromycin, and tocilizumab (NCT04332094); and others. It is evident that combinatorial drug repurposing is a prevalent and likely on the fastest means of identifying a possible effective therapy against the severe acute respiratory syndrome coronavirus 2 (SARS‐CoV‐2) virus/COVID‐19 disease. However, current strategies used to design repurposed drug combinations primarily involve the codelivery of therapies based on the mechanism of action (MOA) followed by dose finding with the objective of finding drug synergy. Unfortunately, drug synergy does not guarantee a successful treatment outcome. This approach, while established, strongly limits the range of drugs that can be considered for use. This is because the addition of more drugs into consideration markedly increases the parameter space that is searchable for optimal combinations. In addition, properly identifying the right doses of each drug in a combination is essential. When both drug selection and drug dosing are considered, sufficiently interrogating the parameter space becomes insurmountable using traditional screening approaches. Of note, the limited sampling of this parameter space, which is virtually assured via traditional combination design and screening, is a core driver of why in vitro results do not translate successfully into the clinic.

Take, for example, a set of 15 candidate therapies against COVID‐19, or any other disease indication. If drug 1 is evaluated against the disease model, and does not exhibit any apparent efficacy, it will conventionally be removed from further consideration. However, it is possible that drug 1 may be a vital component of the drug combination that mediates optimal treatment efficacy and safety. Although a 15‐drug set does not represent a large set of candidate compounds, the parameter space that results from 15 drugs and their respective doses is in fact extraordinarily large. Unfortunately, neither traditional drug screening nor traditional combination therapy design is capable of globally optimal drug combinations from a parameter space of this magnitude. Traditional screening and combination therapy design may also lead to insufficient surveying of a full drug–dose parameter space, resulting in drug combinations that will not comprehensively reflect the unpredictable drug activity or interactions which can be leveraged to globally optimize therapeutic efficacy and safety. In addition, partial drug–dose parameter space screening can lead to the combinations that may exhibit suitable efficacy in vitro that, as previously mentioned, cannot be replicated at the patient level. Unfortunately, this approach can often identify certain drugs or combinations as nonefficacious; in fact, they could be clinically effective while administered in nonobvious combinations at unexpected doses. Therefore, in lieu of MOA‐based drug selection followed by synergistic dose finding, simultaneous drug and dose identification among high‐dimensional search spaces is needed. Although it is well established in drug dosing that dosing impacts drug efficacy and toxicity, it can also determine which drugs belong in a combination therapy regimen in the first place. This is among the most overlooked aspects of drug development. This is a barrier that can be uniquely addressed using artificial intelligence (AI), and, importantly, could enable a markedly enhanced response to COVID‐19 treatment as well as rapid intervention against future outbreaks.

## 2 Accelerating Combination Therapy Development and Optimization with AI

In the context of deploying AI alongside the infectious disease treatment roadmap, it is important to differentiate between the segments of this roadmap. First, the aforementioned use of AI in drug discovery will undoubtedly lead to promising candidates, and these candidates will likely follow a conventional longer‐term drug development pathway toward clinical validation studies. Another segment of this roadmap pertains to drug development, which involves patient matching to trials, and of note combination therapy design.^2^ When rapid intervention is needed during pandemics, for example, AI‐based combination therapy design represents a vital strategy that can identify optimal regimens within a matter of days.

A number of promising methods have previously been explored in an effort to develop infectious disease drug combinations.^3^ Many of the resulting multidrug regimens are based on synergistic drug interactions to amplify treatment efficacy. Synergy can also be used to predict potent drug combinations during the design process. Among all of the strategies that are being used, however, AI‐based platforms have emerged as promising solutions, particularly when they are capable of rapidly optimizing drug combinations without complex disease mechanism data. Project IDentif.AI, a recently developed strategy, does not require big data‐based training sets to rapidly pinpoint suitable drug combinations from extraordinarily large parameter spaces.^4^ Project IDentif.AI is a neural network‐based approach based on a quadratic correlation between inputs defined by drugs and their respective doses and outputs defined by treatment efficacy and safety. This correlation markedly reduces the number of assays needed to pinpoint a list of ranked drug combinations that optimize the treatment outcomes based on a maximized index of efficacy and treatment tolerability. Importantly, Project IDentif.AI can be implemented across the in vitro through clinical phases of drug combination validation. Importantly, the platforms that have served as foundations for the development of Project IDentif.AI have shown prior clinical successes in indications ranging from oncology to infectious diseases. Specifically, positive treatment outcomes were observed in direct ex vivo to clinical validation studies for hematologic cancer (Quadratic Phenotypic Optimization Platform [QPOP]),^5^ and the key role of dynamic clinical dosing on efficacy was further validated on an advanced solid cancer patient (CURATE.AI).^6^ In the area of infectious diseases, low‐dose treatment for human immunodeficiency virus (HIV) was shown to mediate undetectable viral loads while opening the doors to possible downstream reductions in treatment complications arising from side effects of high‐dose therapy (CURATE.AI).^7^

Project IDentif.AI operates in a mechanism and disease indication‐agnostic fashion in that it uses prospectively conducted assays composed of systematically designed drug/dose combinations on a disease model. As such, it does not require training data. This is because these prospectively conducted assays are conducted in a way to effectively survey the full drug–dose parameter space. Importantly, these rationally designed assays define the notion that the way the data are acquired determines our ability to harness AI to optimize treatment as opposed to the sheer volume of data. Once these prospectively executed assays are completed, the corresponding response of the disease model actively drives the optimization process to map out the quadratic correlation from which the optimized combinations can be identified. This is akin to agnostically crowdsourcing the disease model to rank out for us what the best to worst drug combinations are, without prior knowledge of the drug targets being addressed. This is in contrast to traditional approaches which involve validating a set of drugs and set doses. Among such a massive parameter space, there is virtually no way that this strategy can optimize the right drugs and right corresponding doses. As such, AI‐driven drug combination design serves as a potential first line of defense in determining the ideal combination therapies to start with, as opposed to waiting for the results from completed trials to inform subsequent treatment design. AI‐driven combination therapy design may also avoid the use of suboptimal clinical treatments, unnecessary patient mortality, and morbidity, and may even play a role in preventing downstream drug shortages.

## 3 A Globalized Effort to Harness Technology to Optimize COVID‐19 Intervention

In looking at all of the existing/ongoing and upcoming clinical trials against COVID‐19, it is clear that many varieties of combinations have been proposed, and will continue to be proposed as potential solutions. Unfortunately, many of these regimens may not result in positive outcomes because the range of combinatorial permutations and corresponding dosing will ultimately not be assessed to a sufficient degree. A cursory examination of clinical trial databases of the drugs being repurposed, resulting combinations, and dosing regimens reveal that only a fraction of the combinatorial space is being addressed. Although clinical dosing guidelines are well established and are in part guiding the protocols of how these investigational combinations are being deployed, there is an opportunity to harness platforms such as IDentif.AI to substantially increase the degree of actionability of the interventions being developed. When drug combinations are eventually optimized using AI‐driven and related methods, new capabilities will likely emerge. For example, AI‐driven optimization that is conducted during varying stages of infection will likely result in drug–dose compositions and rankings that correspond with each stage. As pathogens evolve over time, the ability to rapidly intervene using AI‐driven reoptimization may also be enabled.

Despite the considerable challenges being encountered with COVID‐19 therapy, the results observed thus far represent an opportunity to unite multinational capabilities in infectious disease drug development with AI to develop broadly deployable regimens for the benefit of the global community. At present, Novartis, Mylan, Teva Pharmaceuticals, and Bayer have collectively committed to donating over 220 million chloroquine and hydroxychloroquine tablets. These efforts have been backed by the manufacturing capabilities of Torrent Pharmaceuticals, Cadila Pharmaceuticals, and Ipca Laboratories, among others. Gilead Sciences has committed to donating 1.5 million doses of remdesivir, and Toyama Chemical has also committed its favipiravir therapy for free to the global community. To complement the trials currently underway, the World Health Organization (WHO) has initiated the SOLIDARITY clinical trial in an effort to facilitate data sharing to accelerate the assessment of treatment outcomes of multiple regimens being studied across the globe. These include local standard of care in addition to remdesivir; chloroquine or hydroxychloroquine; ritonavir and lopinavir; and ritonavir, lopinavir, and interferon beta 1‐alpha. This collection of monotherapies and combinations being assessed represents an ideal opportunity for AI deployment and the systematic derivation of actionably optimized regimens, and can potentially provide a path toward sustained preparedness for future outbreaks and pandemic prevention.

## Conflict of Interest

The author is an inventor of pending patents pertaining to artificial intelligence‐based drug development.

## References

[1] B. Cao , Y. Wang , D. Wen , W. Liu , J. Wang , G. Fan , L. Ruan , B. Song , Y. Cai , M. Wei , X. Li , J. Xia , N. Chen , J. Xiang , T. Yu , T. Bai , X. Xie , L. Zhang , C. Li , Y. Yuan , H. Chen , H. Li , H. Huang , S. Tu , F. Gong , Y. Liu , Y. Wei , C. Dong , F. Zhou , X. Gu , et al., N. Engl. J. Med. 2020, https://www.nejm.org/doi/full/10.1056/NEJMoa2001282.

[2] a) D. Ho , Science 2020, 367, 982; 32108102 10.1126/science.aaz3023

[3] a) A. Zimmer , I. Katzir , E. Dekel , A. E. Mayo , U. Alon , Proc. Natl. Acad. Sci. 2016, 113, 10442; 27562164 10.1073/pnas.1606301113 PMC5027409

[4] A. Abdulla , B. Wang , F. Qian , T. Kee , A. Blasiak , Y. H. Ong , L. Hooi , F. Parekh , R. Soriano , G. G. Olinger , J. Keppo , C. L. Hardesty , E. K. Chow , D. Ho , X. Ding , Adv. Therap. 2020, 10.1002/adtp.202000034. PMC7235487 32838027

[5] a) M. B. M. A. Rashid , T. B. Toh , L. Hooi , A. Silva , Y. Zhang , P. F. Tan , A. L. Teh , N. Karnani , S. Jha , C. M. Ho , W. J. Chng , D. Ho , E. K. Chow , Sci. Transl. Med. 2018, 10, eaan0941; 30089632 10.1126/scitranslmed.aan0941

[6] A. J. Pantuck , D. K. Lee , T. Kee , P. Wang , S. Lakhotia , M. H. Silverman , C. Mathis , A. Drakaki , A. S. Belldegrun , C. M. Ho , D. Ho , Adv. Therap. 2018, 1, 1800104.

[7] Y. Shen , T. Liu , J. Chen , X. Li , L. Liu , J. Shen , J. Wang , R. Zhang , M. Sun , Z. Wang , W. Song , T. Qi , Y. Tang , X. Meng , L. Zhang , D. Ho , C. M. Ho , X. Ding , H. Z. Lu , Adv. Therap. 2019, 3, 1900114.

